# Pocket-size solid-state iPOD and flash drives for gigabyte storage, display and transfer of digital medical images: Review and work initiated

**DOI:** 10.4103/0971-6203.54852

**Published:** 2009

**Authors:** A. Sankaran

**Affiliations:** Ex-Radiological Physics and Advisory Division, Bhabha Atomic Research Centre, Anushaktinagar, Mumbai-400094, India

**Keywords:** Computer/TV image display, flash drive, iPOD, transfer-image devices

## Abstract

A locally assembled image viewer system with pocket-size iPOD (80 GB) and flash (2 GB) drives for gigabyte storage, display and transfer of digital medical images, oriented towards training purposes, is described. Both the iPOD and flash drive enable storage of thousands of images from diverse medical-imaging equipments. The iPOD, in addition, can display with sufficient resolution any of these images and serves as a transportable preview device. Through the use of a computer, these devices can access/ store/ display the images/ photos from a CD, digital camera or the internet. A TV image viewing unit is also provided. The operational features and the advantages of these devices are discussed in detail. The quality assurance (QA) of the displays has been successfully carried out with standard test patterns. The image quality has been tested with dynamic and static medical images. The system will be highly useful for storage and remote display of multitude of images from several modalities in the hospital, as well as other images, from the point of view of education and training. It has good potential for use in clinical diagnosis as well. Other recent advancements using iPHONE and improved but expensive computers, integrated with picture archiving and communication system (PACS) as well as radiology and hospital information system (RHIS) for versatile applications in modern radiology, are also highlighted.This system, assembled with indigenous equipments, is much less expensive and specially suited for teaching radiologists, physicists and technologists, particularly in developing countries.

## Introduction

In the field of diagnostic radiological imaging, the transition from analog to digital was closely followed by the development of the picture archiving and communication system (PACS).[1–3] Concomitantly, multidimensional imaging techniques [4D and 5D, for motion (temporal) and functional (fusion image) studies on 3D images, respectively] have presented new challenges, particularly in handling gigabyte-size images from CT, MRI and PET scanners, which generate thousands of images. The storage and analysis of these images necessitates expensive image workstations. The use of the iPODs and flash drives for storage of gigabyte-size images has been investigated by several medical institutions worldwide. The motivation for this paper originated from the work accomplished in the Department of Radiological Informatics, UCLA (USA), where an educational software OSIRIX[4] (this reference comprises the latest exhaustive literature on the subject) was developed for acquiring/ displaying/ analyzing images from various imaging modalities, based on standard digital imaging and communications in medicine (DICOM) protocols. This open-source DICOM-compliant software is available for free downloading but was written for use with the Apple Mac Os X computer system. (Windows-based softwares for accessing images from DICOM files are available from other vendors.) The versatility of this software when used with a Mac computer is succinctly described in a tutorial booklet.[5] The researchers in this institution (UCLA), in conjunction with this development, have implemented several innovations in the use of the iPOD system for medical imaging education. (The websites on the use of the iPOD and flash drive for medical education and training are very extensive.) Although the potential uses of the iPOD were recognized, it was realized that the iPOD display at best serves only as a small-size viewer, and the associated Microsoft Windows home PCs and TV display systems used along with this system may not be suitable for detailed analysis, advanced image processing and interpretation of images necessary for medical decision-making for which expensive image processing workstations are still required.

Radiology and imaging industry personnel in the US, along with Apple Company, have recently come out with a cost-effective solution to this problem and have recommended a medical imaging Mac OS X-based system integrated with the OSIRIX application. Windows-based systems can operate within this system with potential advantages. The requirements for, and the full description of, this computer system with its features and advantages are vividly described in an excellent white paper.[6] This system can be integrated with the PACS, radiology and hospital information system (RHIS) and teleradiology as well. Apple has also recently released into the market a versatile pocket iPHONE 3G[7] which combines the features of a revolutionary mobile phone, a widescreen iPOD and a breakthrough internet device with rich HTML email and a desktop class web browser. The imaging software for this system is being continuously developed by Apple and other companies in US. This is anticipated to provide a better DICOM viewer (perhaps a possible mobile workstation) along with Wi-Fi and other features for telemedicine compared to the iPOD; but some concerns from hospitals, such as security, confidentiality and patient privacy, in using these devices from the point of view of patient data storage and diagnosis have to be addressed before these devices are put to clinical use.

All the above revolutionary, progressive developments, in spite of their utility, are expensive and difficult to implement in radiology departments of developing countries lacking funds, expertise or infrastructure. The use of the iPOD, flash drive and other relatively less expensive USB storage devices for transport of data, including imaging data, although widely known and investigated in advanced countries, has not still made much impact in developing countries. There is a need to evaluate these devices independently and highlight their applications, particularly from the viewpoint of education and training of radiologists, medical physicists and technologists in this part of the world. Besides imaging from several modalities, other subjects such as anatomy, radiology, medical physics equipments and their QA, medical procedures, etc., can also be efficiently taught through these media. The potentialities of these devices with the associated computer/TV displays for clinical diagnosis remain to be fully explored. This article is a sincere attempt in this direction and also serves to explain their salient operating features and merits and demerits to new users from the point of view of medical imaging. The article also illustrates how various locally available equipments (some of them are already used in everyone's home) can be readily assembled together to form a low-cost viewer system for medical and other imaging studies using these devices (presently used primarily for entertainment and other data storage purposes) along with other input devices.

Full technical details and characteristics of the iPOD and flash devices and their advantages over conventional storage devices are accessible from several websites through GOOGLE search engine. Basically, flash drives and iPODs of moderate GB storage capacity are based on EEPROM (electrically erasable programmable read-only memory) solid state technology, whereas iPODs of large GB storage capacity utilize hard disk drives. These devices are superior to conventional storage systems such as floppy diskette, R/W CD, zip drive, etc., in view of their extremely high storage capacities, ruggedness and fast read/ write/ erasure cycles. Recent iPODs are available with memories up to 160 GB (120 GB is more popular), whereas flash drives have a much lower storage capacity (up to about 64 GB). It may be noted here that BluRay discs can store easily up to 50 GB and up to 200 GB with multilayering, and these devices also can be used for short-term image storage. The flash drive is a miniature programmable nonvolatile data storage device, while the iPOD serves, in addition, as a pocket audio/video player (with the capability of storage of thousands of images and audio/videos) and displays video images/photos on its screen.

## Materials and Methods

**Figure 1:**
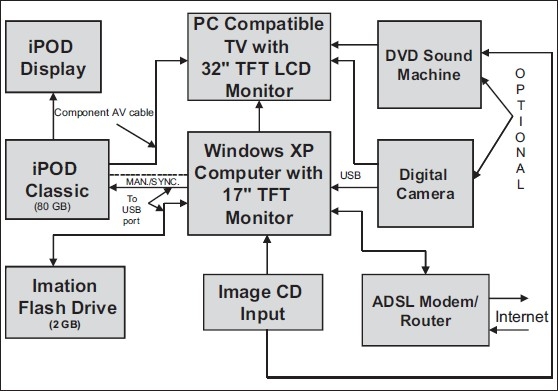
Block diagram of setup for image/ photo viewing from flash drive and iPOD, as well as from CD, digital camera and the internet

A 2-GB swivel flash drive (also known as pen drive, jump drive or memory stick) (Imation Europe BV) and an 80-GB iPOD Classic (Apple Inc., California) were procured for this study. The salient features, and requirements for use of these devices are set forth in Tables 1 and 2, respectively. These devices are less expensive as compared to the iPHONE and iPOD TOUCH devices (marketed by APPLE), which also have been investigated by other workers for imaging purposes. The most recent work is the OSIRIX on iPHONE, which can directly receive images from any DICOM imaging device remotely through the Wi-Fi network and manipulate them. It is worth noting that the flash drive includes a program to create partitions on the drive and to set a password-protected secure area. If required, the device may also be write-protected. This will be of use for storage of special patient image data. Somewhat similar to this feature, a digital locking facility is provided in the iPOD for prevention of misuse of the device by unauthorized users. Both the flash drive and the iPOD can also be employed for storage of many text files needed for education. But the iPOD, on its own screen, can display only a small text file (.txt) under the NOTES folder. PDF and MS word files have to be converted to .txt file for viewing this display. For imaging studies with emphasis on education and training using the flash drive and iPOD, the author, with his own meager resources, has locally assembled a prototype, which is a simple, general-purpose, experimental image-viewing setup [Figure 1] using indigenous equipments. The images/photos accessed by these devices from the CD, digital camera (see below) or the internet can be displayed on the computer or TV monitor. The accompanying audios (e.g., in some demo programs) can also be played simultaneously. A PC-compatible TV with 32 inch color thin film transistor liquid crystal display (TFT LCD) monitor (Samsung model 3 series, Samsung Worldwide, available at TV showrooms in Mumbai) was used to display the images from the iPOD and digital camera and to play videos/audios from the DVD sound machine (see below). The color TV display has a screen resolution of 1360 × 768 pixels and is provided with remote adjustments for contrast, brightness, sharpness, zoom, color, gamma, edge enhancement, digital noise reduction (DNR), etc. The computer used was the latest (model Wipro WIV15D55, Wipro Limited, Bangalore, India), provided with cyberLink DVD suite, NERO StartSmart essentials (with features to copy/erase CD/DVD, burn image to disc, etc.). The 17-inch TFT LCD monitor (model 716Swx, also from Wipro Limited, Bangalore, India) has a maximum screen resolution of 1440 × 900 pixels and a color quality of 32 bits. The Microsoft Office Picture Manager (part of Microsoft Office) in the computer has the facility to edit pictures using tools such as brightness and contrast, color, crop, rotate and flip, change picture size and an auto-correct facility to automatically correct color and brightness. All these TV and computer features facilitate obtaining pictures of optimum quality.

**Table 1:** Features and characteristics of Imation Swivel USB 2.0 flash drive*

Utilizes EEPROM technology. Portable Plug and Play connection. Enables exchange of text, video and photo files easily between computers with a USB port. High storage capacity (2 GB) and fast speeds. Miniature portable device – replaces floppy drives, ZIP drives and hard drives. Includes a program to create partitions on the drive and set a password-protected secure area for personal/ image data. Imation Drive Manager.exe used to format/ partition the USB flash drive and set security area. Login.exe used to access the security area. Compatible with desktop or laptop computers with USB 1.1 or USB 2.0 port. Powered from USB port, no external power or battery needed. Multiple operating systems supported – no driver needed in Windows^®^ ME, Windows^®^ 2000, Windows^®^ XP, Mac TM 9.x or later versions, Linux TM Kernel 2.4 or later versions. Only Windows^®^ 98SE requires a downloadable driver – installable from the Imation website http://www.imation.com/products/flash_devices/download.html Secure removable mass storage for scientific, medical and personal data use. Write protection provided. Shock resistant, noise free; with long-term data retention.

* Adapted from Imation flash drive user's manual and Imation website (for educational use)

**Table 2:** Features and requirements of iPOD classic*

*Features*
Device Type: Portable media center. Digital audio and video player/ photo viewer. 4.1 inches × 2.4 inches × 0.4 inch; weighs only 4.9 oz. PC Interfaces: USB 2.0. Digital Storage Capacity: 80 GB. Digital Storage Type: Hard disk drive. Display Type: LCD 2.5”. Supported Still Image Formats: BMP, JPEG, PNG, GIF, TIFF, PSD, etc. Supported Audio Formats: Protected AAC, Audible 3, WAV, MP3, VBR, Audible 2, AIFF, MP3, AAC, Apple Lossless, Audible 4. Audio Output: headphones, mini-phone stereo 3.5 mm. Video Playback Formats: MPEG-4 Video Capture: 640 × 480 pixels^1^. Battery: Lithium ion rechargeable player battery (integrated); recharge time: 4 hours. Battery Life Details: Audio playback 30 hours, video playback 5 hours; battery life: 30 hours. Software Included: iTUNES 7.4 or later version, drivers. Accessories Supplied: Docking adapter, USB cable, earphone.
Requirements
Operating System: Apple Mac OS × 10.4.8 or later version; Microsoft: Windows XP Home, Windows Vista, Windows XP Professional SP2^2^ or later version; broadband internet connectivity recommended. Other Optional Downloadable Softwares: Quick-time player, Jodix video converter, Videora/Total video converter, E-Zsoft iPOD converter, PDF to .txt converter. Component AV cable to connect to TV^3,4^. iPOD Display Resolution: 320 × 240 pixels. Latest model with cyberLink DVD suite, NERO StartSmart essentials and broadband internet, used by the author. Latest TV model with 32” LCD monitor, used by the author. Supplied with charger unit for charging the battery during continuous use.

* Adapted partly from iPOD user's manual and specifications from Apple websites (for educational use).

### Peripherals/ Enhancements and additional features

The author, based on his earlier teaching/ R & D experience, has found that some of the images very useful for medical imaging education can be obtained by on-the-spot photography (e.g., those of medical imaging/ therapy/ nuclear medicine units and associated medical procedures, QA gadgets and test equipments, QA and calibration/ test procedures, to name just a few) in a medical institution or research center and transferred later to the iPOD or flash drive for teaching purposes. Apart from this, a modern digital high-resolution camera, being compatible with the TV and computer, will be a valuable tool for checking/ comparing the image quality of these displays with the photos (e.g., that of a transparent/ translucent contrast detail phantom) produced by it. Therefore, a high-resolution digital camera was added as an optional accessory. The digital camera employed was a pocket-type charge-coupled device (CCD) high-resolution (10-bit pixel resolution, 3264 × 2448 pixels) device (model Finepix J10, Fuji Film Corporation, Tokyo, Japan) with amorphous silicon TFT color LCD monitor. It is provided with USB and A/V cables for connection to a computer through the USB interface and to a TV display, respectively. A. movie-recording (with monaural sound) facility with a movie file capacity of about 2 GB is also incorporated. Another peripheral that the author has included for teaching implementation (preparation of modules and lectures) is the DVD sound machine (Philips Model AZ5737, Philips, Australia/China, available at electronics shops in Mumbai, India). It combines a DVD player, FM/ MW radio and audio cassette player/ recorder in one compact unit. (Small cheaper versions having only a DVD player, available locally, can also be employed.) This will be suitable for directly playing audio/ video CDs containing instructive materials, educative literatures from companies; audio capture of lectures by experts, etc., on the TV/ computer system and/ or importing them later to the iPOD or flash drive storage for teaching. This item is added in view of its versatility in playing several types of CDs (DVD, VCD, SVCD, MP3/ JPEG picture disc, etc.). Also, it is compatible with all commonly used audio/ video codes. Generally, in all modern computers (as the one used by the author), the DVD player forms part of the computer system, and a separate DVD player may not be always required.

### Technical details and operational/ display features of the devices

The technical description and detailed User's Manual of the flash drive are accessible from websites[8,9] or by opening its software contents. The flash drive is supported by multiple operating systems [Table 1]. For operating with the Windows 98SE system, however, the driver software is required. Following the instructions in the Imation Flash Drive manual, the author succeeded in interfacing the flash drive to the Windows 98SE system (earlier used by him) by downloading and installing the software from the link Windows 98 SE Generic USB Mass Storage Device Drivers v3.3 – nusb33e.exe. or directly from Imation website [Table 1]. However, iPODs are not compatible with the Windows 98 system. (Table 2 for computer requirements for the iPOD.) The author, therefore, replaced this old system with a new more versatile Windows XP Professional system provided with a 17-inch TFT LCD monitor [Figure 1] and various built-in features [Table 2]. In India, modern computer systems are freely available at increased cost, but the old Windows 98 systems still continue to be used.

The iPOD classic is compatible with both the Windows XP and the Windows Vista systems and hence does not require any additional driver software for interface. For transfer and storage of images from these modern computer systems to an iPOD, it is first necessary to download and install the free Apple software iTUNES,[10–12] along with other utility software [Table 2]. (The iTUNES software is the heart of the iPOD. Full details can be had from the iTUNES tutorial, which is part of the iTUNES.) The computer can then directly import video images, audios, and photos from CD, digital camera or the internet into the iTUNES library, display them on its monitor and then transfer them to the iPOD. The iPOD serves as a portable alternative to a conventional DVD player with a TV/ computer display, with extremely high storage capacity. For import and transfer of image files to the iPOD, file format compatibility must be ensured. This iPOD can receive/ play images in MPEG-4 and H.264 (video) files only.[10] (See supported video and audio formats in Table 2.) For playing audios/ videos from noncompatible files, software conversion for creating the iPOD version can be effected with a converter already built into the iTUNES software. Several audio/ video converters (as well as text converters) are also available for free downloading from the internet [Table 2]. The author has utilized these converter softwares with success. The audios, video images, etc., from a CD or the internet imported to the iTUNES library can, after code conversion, be transferred to the iPOD only through a computer. Photos from a digital camera or those imported from the internet in JPEG and other compatible formats (JPG, BMP, TIFF, PICT, GIF, TIF, PSD, SGI and PNG) into the picture folder can be similarly transferred to the iPOD.

**Figure 2:**
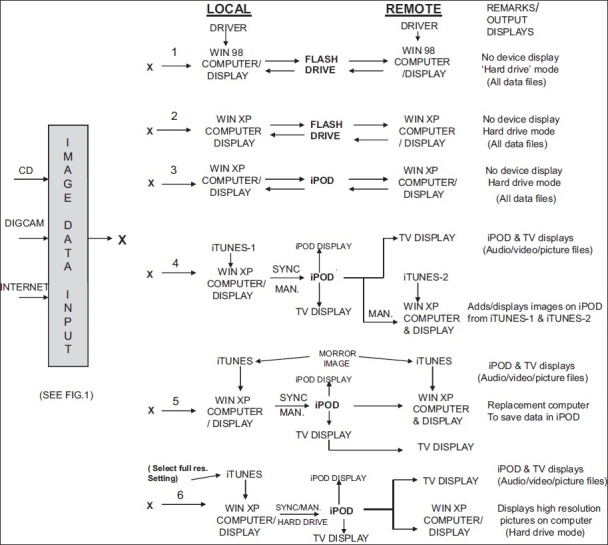
Different configurations for operation of flash drive and iPOD devices

Figure 2 vividly illustrates the different configurations for the operation of these devices (Apple 80-GB iPOD classic and Imation 2-GB Swivel drive), indicating the various ways and steps to transfer the image data to the flash drive and iPOD and to display them on local and remote units. As an aid to new users, a brief description of each configuration is provided below:

1. This depicts the least expensive viewer using the Windows 98 system with flash drive where a built-in image device display is not a requirement. As stated earlier, this requires downloading and installation of the driver software. All files (audio/ video, picture and text files) can be transferred/ stored in the flash drive and displayed on both the local and remote computer.
  All the remaining configurations described below utilize the Windows XP computer system for local displays; and for remote displays, where applicable.
2. The steps and displays are the same as those for (1) above. Since this computer is compatible with the flash drive, the driver software can be dispensed with.
3. This configuration [functionally same as (2) above] uses the iPOD as a hard drive with local and remote computer displays (no device preview display). This is recommended when greater storage capacity of the iPOD is necessitated.
4. In this configuration, both SYNC and MANUAL modes are used to transfer image data to the iPOD through the iTUNES/ iTUNES library. In the SYNC mode, the audio/ video files imported to the iTUNES-1 library and photos in the picture folders are synchronized with those in the iPOD in the local computer; whereas in the MANUAL mode, any selected audio/ video file in iTUNES-1 library or pictures from folders can be manually transferred to the iPOD source in the iTUNES library. Audio/ video/ picture images thus transferred in SYNC and MANUAL modes can only be displayed on the iPOD screen or on a TV system (local or remote) connected to it, for listening/ viewing. The data transferred manually to the iPOD can be displayed on the remote computer, from where additional images/ pictures from the iTUNES/ iTUNES-2 library can also be MANUALLY added to the iPOD. If the iPOD is connected to the remote computer in SYNC mode, its contents will be replaced by contents of iTUNES-2. Information pertaining to the file (audio/ video) or picture folder can be entered in both the computers prior to transfer.
5. This scheme resembles (4) above except that the iTUNES library and picture folders in the local computer are initially copied to the remote computer (iTUNES 1 = iTUNES 2), the remote computer thus acting as a mirror image of the local computer. This type of remote display operation is the preferred choice. Moreover, in case of local computer failure, the remote computer serves as a standby for the preservation and display of data stored in the iPOD for further use of the iPOD.
6. In this configuration, the ‘full resolution’ setting for pictures in the iTUNES is selected. This enables storage of the original full-resolution images in the iPOD hard drive and their display. Normally, when these full-resolution pictures are transferred to the iPOD in the other modes mentioned above, the pixel resolution of the picture is optimized for the iPOD display only.

It may be added here that for all the remote computer displays not accompanied by TV display facility, one can always use a computer-compatible TV system, as used by the author. Note that full-resolution pictures are made use of for all TV displays.

To summarize the steps mentioned above, the transfer from a computer to the iPOD is a one-way process (from computer to the iPOD only) when using SYNC and MANUAL mode of operation. In the disk-mode operation, for both the iPOD and the flash drive, it is a two-way process (computer to the device and device to the computer). These are shown by suitable arrows in Figure 2. It must be stressed here that, for the trained user of iPODs, selection and application of these techniques of operation for the desired output display comes automatically. They do illustrate the versatility of operation of the iPOD.

It may be noted that the iPOD display, though small in size with reduced resolution [Table 2], serves as a quick medium-quality previewer. For better-quality viewing, a high-resolution computer or, better still, a 32” TV display with a pixel resolution of 1360 × 768 or higher should be employed. Apple provides a special component AV cable (with connections to the built-in iPOD battery charger) for connecting the iPOD to a TV set for long-term viewing. Since most of the softwares which require downloading/ updating (e.g., iTUNES, PC security, utility softwares) occupy large storage space (tens of megabytes), a broadband internet connection is recommended. (The dial-up system takes hours for downloading.) The author therefore changed his telephone line from dial-up to the high-speed MTNL (Mumbai telephone authority) triband (250 bps – 1 Mbps) using a locally available modem (ADSL2 + Router, manufactured by D-Link India Limited, Goa, India). For details, see Figure 1 and Table 2. ADSL (asymmetrical digital subscriber line) is the transmission technique used on the line from the modem to the service provider. It provides simultaneous availability of the phone and internet on the existing telephone line (“Always on Internet”). This is particularly useful for quick access of any website and faster downloading as compared to the dial-up system. Parallel telephone lines can also be simultaneously used for conversation without disturbing the internet browsing/ downloading/ chat activities. This capacity could be useful during remote training, allowing the student to converse with the instructor (or interaction between two medical professionals while discussing a case) while simultaneously downloading applicable images.

### Quality assurance of displays

Objective testing of image quality of display systems by a single observer can be done by using the test patterns. Perhaps the best and latest among them is the PACS test pattern in digital format, available commercially (e.g., Nuclear Associates 07-450-4000 PACS Digital Test Pattern loaded in CD). As this is expensive and time-consuming to import, the quality assurance (QA) of the iPOD and the TV/ computer displays can be alternatively carried out with the widely used standard SMPTE (Society of Motion Picture and Television Engineers) medical imaging test pattern and TV color bar pattern generators.[13] (The contrast detail phantoms used by physicists serve to check the overall image quality, including that of display.) The SMPTE pattern contains low- and high-contrast–resolution targets, a grid for distortion measurement and a gray scale. The pattern also includes low-contrast areas in the dark and light areas of the scale to allow a quick visual check to determine if the entire video signal is visible in the video display. This test should be done ideally by company engineers for commercially available systems. As there was no reason to doubt the image quality of the displays (all the units were purchased only recently and because of the good image quality obtained by the author earlier for several images), getting this testing done by engineers was not attempted. Instead, the author downloaded the two SMPTE test patterns and additionally a test CT image (showing a typical pancreas carcinoma with all surrounding organs) from suitable websites and displayed them on the computer; and after transfer, displayed them on the iPOD and TV. Recently, a Rubo Dicom Viewer[14] (an improved color version of SMPTE test pattern) has become available for free downloading for trial testing of displays. As this pattern was ‘read-only’ and write-protected, tests could be/ were done with this on the computer display only.

## Results and Discussion

Both the iPOD and flash drive systems were satisfactorily tested for storage and faithful reproduction of dynamic ultrasound and x-ray images earlier obtained in 2D-echo and angiogram diagnostic procedures on a patient and stored on compact discs. These images being dynamic were imported to the movies folder in the iTUNES library and transferred to the iPOD [configuration 4, Figure 2]. In case of the flash drive, these files are dragged on the I:drive icon under ‘My Computer’ [configuration 1 or 2, Figure 2]. Typical CT, MRI and PET scans; fused images and photos related to patient diagnosis (static images); etc., as mentioned earlier, were also imported from suitable educational websites to the picture folder in the iTUNES and then transferred to the iPOD and displayed on the iPOD display (configuration 6). All the transferred files are still preserved with the original pixel resolution in the computer and iPOD, and any of them can be displayed and viewed on the computer display with good clarity and crispness. Despite its small size, the video images displayed on the iPOD were of sufficiently good quality, indicating that iPOD can be used as a nice medium-quality previewer for educational purposes. The same images/ photos were also displayed on a TV to which the iPOD was connected using the component AV cable. Since the TV has a resolution of 1360 × 768 pixels, the original images/ photos must have the same or better resolution, or else the pictures will be blurred. However, for the iPOD display, the same picture is optimized to a reduced picture resolution (640 × 480 pixels). The combined audio/ video images (typical education demo softwares, e.g., pertaining to new products and equipments, radiology teaching, etc.) downloaded from the internet or imported from CDs and transferred to the iPOD, were also displayed on the computer, TV and iPOD. Similarly the audio quality alone was separately tested with the audios already stored in the iPOD. Both the video and audio quality turned out to be very good on both the systems, showing the versatility of the iPOD. To test the effect of pixel resolution, some still pictures were taken with the digital camera using the high pixel resolution of 3264 × 2448 pixels, transferred to the iPOD in SYNC mode [configuration 6, Figure 2] and displayed on the computer, iPOD and TV displays. The computer and TV display pictures had really impressive crispness and clarity. The camera and iPOD displays, though small and same in size, were equally clear and comparable. Also, some clips of cine movies earlier transferred from the DVD and stored by the author were also displayed/ played on the iPOD, with excellent clarity. Note that for the computer and TV displays, the picture quality was optimized using the features mentioned earlier. It must be pointed out that since the iPOD and flash drive are basically storage systems, the images transferred retain their original pixel resolution (number of pixels) and pixel depth (gray level) characteristics without loss.

### Quality assurance tests on displays

The following visual checks were done on the iPOD, computer and TV displays and verified fully, with the SMPTE test pattern[13] (512 × 512 pixels):

1. The 5% patch was clearly visible inside the 0% patch.
2. The area of the 0% patch was almost black with raster lines not visible.
3. The 95% patch was visible inside the 100% patch.
4. The alphanumerics were sharp and clear.
5. All the horizontal and vertical high-contrast/ low-contrast line-pair patterns at the five different areas (middle and four corners) were fully resolved.
6. There was absolutely no distortion or blurring on any portion of the displays.

The above requirements form some of the criteria recommended for QA of displays by AAPM.[15] As stated earlier, these constitute objective testing of image quality of display systems by a single observer.

For achieving good results, picture quality adjustments were made using the features in the computer and TV, mentioned earlier. The best processed image obtained on the computer display was transferred to the iPOD for comparison. Similar tests were carried out and corroborated fully with the SMPTE color bar pattern (180 × 135 pixels), i.e., all the different colors with patterns were clearly visible without any distortion of the edges on the iPOD, computer and TV displays. Also, the test CT image (640 × 480 pixels) clearly displayed the pancreas carcinoma with clear delineation of all surrounding organs.

The iPOD preview display compared very favorably with the other displays for both test patterns and the test CT image, in spite of its reduced size. These tests conclusively proved that the iPOD as a previewer and the associated TV and computer systems are quite adequate for display of images with sufficient pixel and gray level resolution without distortion and loss of information, although one would have expected some loss of details via the internet. These tests are very important for deriving correct diagnostic information from the TV and computer displays for imparting education to trainees and for possible extension to clinical diagnosis.

Tests (1 to 6) mentioned above were also corroborated with the Rubo Dicom Viewer pattern (this has high- but no low-contrast–resolution line-pair patterns) on computer display. In addition, all the colors were fully resolved.

**Figure 3:**
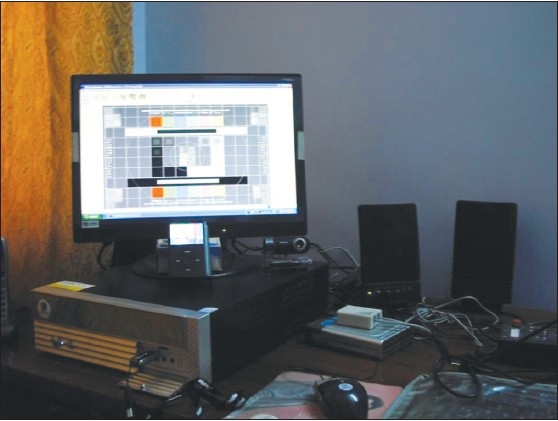
Photograph of the image viewer, showing the computer with associated parts; also, iPOD and flash drive. The computer displays the Rubo Dicom Viewer test pattern (see text)

**Figure 4:**
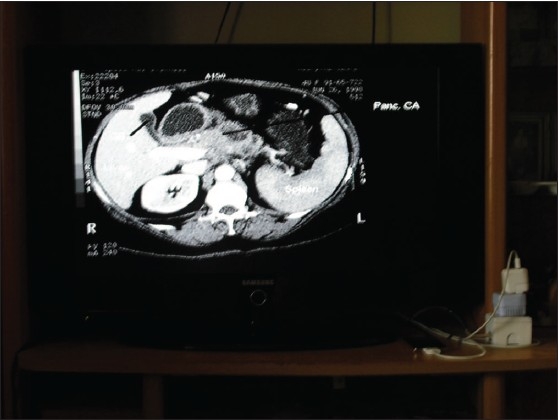
Photograph of the image viewer showing the iPOD connected to a TV display. The TV displays the test CT image (see text)

The photographs [Figures 3 and 4] taken with the camera show the different parts of the image viewer system set up in the author's home. In Figure 3, the iPOD display, the flash drive connected to the computer, the modem, web camera and the stereo speakers are clearly visible. The TV system connected to the iPOD with its charger on the right can be visualized in Figure 4. Typical images (Rubo Dicom Viewer pattern and the test CT image, mentioned above) are displayed on the computer and TV, respectively. For viewing on a remote site, the high-resolution images taken with the camera were later compressed (to about one tenth of the original size) for quick transmission.

### Note on the preservation of data stored in the devices

A note on the use of these storage devices in education and training is now appropriate for new users, as well as for medical professionals. While the data storage and operating life of these flash drive and iPOD devices are comparable to those of other solid-state storage/ hard drives, respectively, in practice, in view of these being used here predominantly for storage of many medical images, sufficient back-up systems must be used. The necessity arises because, as compared to other storage devices, these devices are more susceptible to other problems (from as simple as loss/ breakage to unintentional reformatting and virus infection, particularly compared to write-protected DVD). An instructor or a medical professional who has imported many medical images from several sources and transferred them to the iPOD or flash drive will find it difficult to retrieve them again once they are lost. High-capacity write-protected DVDs (which are relatively less expensive) can be successfully employed as back-up systems. As mentioned earlier, the computer system used by the author has the Nero StartSmart essentials facility to burn the image data to the DVD. The requirement of back-up systems is more pronounced in case of iPODs since a crash of either the computer or the iPOD may result in the complete loss of these images. In case the computer is functional and if the iPOD images are lost (due to corruption of memory, virus or software problems) and if the original images are preserved in the iTUNES library, it is still possible to transfer the images to the repaired iPOD (or, if it is completely damaged, to a new iPOD); if the computer is damaged, it is also possible to connect the iPOD to a remote TV or another computer and display the images. As pointed out earlier, display on another computer will be possible, provided the images were earlier stored in, or transferred to, the iPOD in the MANUAL or hard-disk mode. All the video images earlier stored in the SYNC mode will, however, be lost unless a mirror image of the entire iTUNES library of the first computer had been created earlier in the second computer (see configuration 5, Figure 2), which can be easily accomplished. [In this context, it is pertinent to bring to the attention of the readers that a new iPOD transfer wizard software[16] (‘MediaWidget’) for Windows has been just introduced to solve some of the above iPOD problems. Besides retrieving the data in the event of a computer or iPOD crash, this software enables easy to-and-fro data transfer between the computer and the iPOD.] To avoid corruption of image files by virus, spyware, firewalls, etc., the author has installed a highly powerful internet security system in the computer (Trend Micro, available through the Tata Indicom server, Mumbai). The flash drive is a passive storage device, and data transfer to and from the computer is established through a USB port. The iPOD battery has a finite life, similar to other chargeable batteries undergoing many charge and discharge cycles. The battery is automatically charged when the iPOD is connected to the computer through a USB port. As stated earlier, an external charger is part of the component AV cable. For battery replacement, Apple advises clients to get this done through their agents located in important cities of all countries, including Mumbai. All maintenance and precautionary instructions, as well as trouble-shooting tips, on the iPOD are available from the user's manual, Apple website and the Apple Support Team[9–12] for Apple clients.

## Conclusions

Both the iPOD and flash drive systems serve as handy, pocket-size, ingenious devices for mass storage and transfer of medical images for education and training and possible clinical use. In addition, the iPOD, although basically a music player, enables medical professionals to easily transport and view medical images (also on a TV or computer screen) at any site inexpensively. This facilitates mutual consultation/ discussion between radiologists or members of the medical team about any specific case. Useful peripherals have been added as enhancements of the viewer. The cost of various components of the associated image viewer system, assembled with readily available indigenous equipments, is given in Table 3. If the optional accessories are excluded, the actual cost is 78345/- Indian Rupees (about $1600), which is optimum considering the various computer/ TV features offered. Also, for possible general diagnostic applications, this cost is quite low compared to the expensive workstations. The display pixel and gray scale resolutions obtained are more than adequate for these purposes. The importance of this viewer for developing countries cannot be overemphasized. The OSIRIX educational software for viewing the DICOM image files for Windows PC will soon be installed and tested. The MediaWidget software is already installed and will be an added feature. The audios/ videos stored in the iPOD and pictures stored in folders/ sub-folders can be identified alphabetically. The Adobe Photoshop 7.0 software has been downloaded and installed for better manipulation and editing of still images. Both the devices have built-in security features as mentioned earlier, required for possible clinical applications. The operation of the system has been reliable and consistent during the past several months.

**Table 3:** Cost of the system for image/ photo viewing@ from flash drive, iPOD, CD and digital camera [Figure 1]

	*Cost in US dollars*	*Cost in Indian rupees (Rs.)*
iPOD Classic, 80 GB (Apple model)¶	($250)	12250/-
Component AV cable (Apple)	($25)	1225/-
Flash drive, 2 GB (Imation model)	($30)	1470/-
Computer (Windows XP with 17” TFT monitor) (Wipro model)*£		24000/-
TV set with 32” LCD monitor (Samsung model)*		38000/-
DVD sound machine (Philips model)*#		5900/-
Digital camera (Fuji film Finepix J10 model) #	($150)	7350/-
ADSL modem/router (D-link model)*		1400/-
Total cost: I. Rs.		91595/-

Notes:

* 1. Items normally used locally for scientific/ education/ home entertainment purposes.

2. Prices are approximate.

# 3. Optional: Camera for photography (including audio/ video) in connection with education (see page 6).

4. All models as used by the author; other equivalent models will also serve the purpose.

5. Prices in Indian rupees: US$1 = I. Rs. 49/- approximately. Also, prices of imported items in US dollars.

¶ 6. Normally used as a music/ movie player, for entertainment purposes.

£ 7. Excludes cost of licensed software CDs for Windows XP, Microsoft Office, internet security and other utility software CDs; to be purchased separately.

@ 8. Not meant to replace imaging workstations.
